# Declines in Electronic Cigarette Use Among US Youth in the Era of COVID-19—A Critical Opportunity to Stop Youth Vaping in Its Tracks

**DOI:** 10.1001/jamanetworkopen.2020.28221

**Published:** 2020-12-01

**Authors:** Andrew C. Stokes

**Affiliations:** School of Public Health, Boston University, Boston, Massachusetts.

The use of electronic cigarettes (e-cigarettes) has increased at an alarming pace among youth, a striking reversal of decades of public health progress aimed at reducing youth tobacco use in the US. Between 2011 and 2019, the proportion of high school students who were current e-cigarettes users increased from 1.5% to 27.5%.^1^ Any use of e-cigarettes by youth is unsafe and includes short- and long-term health effects, such as impacts on the brain, lungs, and heart.^2^ The rapid uptake of e-cigarettes in the youth population is likely attributable to a number of factors, including the appeal of flavors, technological refinements in the design of e-cigarette products, and ease of access to vaping products by underage youth despite existing regulatory policies.^3^

The coronavirus disease 2019 (COVID-19) pandemic has had far-reaching consequences since emerging in the US in February 2020. This new study by Gaiha and colleagues^4^ examines the associations of the COVID-19 pandemic with patterns of youth e-cigarette use, drawing on a national sample of 2167 underage youth and young adults sampled between May 6 and 14, 2020. Among the key findings of the study were that more than half of participants (1198 [56.4%]) reported change in their e-cigarette use since the beginning of the COVID-19 pandemic. Among 1197 participants reporting on the type of change, one-third of youth (388 participants [32.8%]) quit vaping, and another one-third (422 participants [35.3%]) reduced their use of e-cigarettes, with the remaining youth either increasing their use or switching to other nicotine or cannabis products. Second, the point of purchasing e-cigarettes changed markedly before and after the COVID-19 pandemic, with approximately 20% of youth e-cigarette users switching from retail stores to online sources. Third, the study examined factors associated with continuing to vape through the pandemic, finding that higher nicotine dependence, e-cigarette use frequency, poor online age verification, and several other factors were associated with sustained use of e-cigarette products.

The findings in the study by Gaiha et al^4^ of substantial declines in e-cigarette use associated with COVID-19 is consistent with other emerging evidence. Data from the 2020 National Youth Tobacco Survey (NYTS)^1^ indicate that approximately 20% of high school students and 5% of middle school students were current users of e-cigarettes in early 2020, compared with 27.5% of high school students and 10.5% of middle school students in 2019. These data, collected between January 16 and March 16, 2020 suggest that substantial declines in e-cigarette use may have occurred even prior to the period of the study by Gaiha et al^4^ and in some cases before stay-at-home orders came into effect.

Among 895 participants who decreased or quit use of e-cigarettes, Gaiha et al^4^ inquired about the reasons for decreased use. Explanations for decreased use included at home and parents will know (136 participants [15.2%]), cannot get products (175 participants [19.5%]), e-cigarettes may weaken lungs (224 participants [25.0%]), any combination of these factors (287 participants [32.1%]), and other (73 participants [8.2%]). These breakdowns point to the roles of both supply-and demand-side factors in shaping patterns of e-cigarette use during the COVID-19 pandemic. With respect to the supply side, reduced access to products was an important factor, and on the demand side, participants’ perceptions regarding health consequences of e-cigarette use appeared to change (eg, that e-cigarettes may weaken lungs). The fact that one-quarter of youth reported concerns regarding health effects of e-cigarette products as a major motivating factor for quitting or reducing e-cigarette use indicates that targeted educational campaigns may be a fruitful strategy for further reducing youth vaping. Such efforts may be made especially compelling by communicating potential associations of vaping with incidence and fatality of COVID-19, a link that is supported by emerging literature, such as another 2020 study by Gaiha et al.^5^

In this study, Gaiha et al^4^ also reported that purchasing of e-cigarettes shifted increasingly online. This is consistent with emerging qualitative evidence by Schiff et al^6^ that online retailers are frequently accessible to underage youth and that even when age verification is in place, it is often easily circumvented. The shift toward online purchasing highlights a critical need for the Food and Drug Administration to use its regulatory authority to prohibit online sales to underage youth and requiring a strict burden of proof by e-cigarette companies of their age verification systems at the time of applying for market authorization.

Perhaps not surprisingly, youth with more frequent e-cigarette use and higher nicotine dependence were more likely to continue vaping during the COVID-19 pandemic.^4^ These findings may in part reflect the lack of comprehensive addiction services for youth who vape or a lack of awareness of existing services. Additional research is required to understand the effectiveness of different approaches to addressing youth vaping addiction, including programs using novel approaches, such as social media, virtual reality, and text message programs. Further work on how to deliver these programs at scale, given the magnitude of youth vaping in the US, is also needed.

The results reported by Gaiha et al^4^ indicate that reductions in youth vaping were not greater in underage youth compared with older youth who were aged 21 years and older, despite underage youth having experienced greater restrictions on use in their home environments. In fact, the least cited reason for decreased use of e-cigarettes during the COVID-19 pandemic was that parents will know, suggesting an ability to use e-cigarette products discretely, even in the home environment. As Gaiha et al^4^ point out, these findings point to a critical need to regulate how e-cigarettes are designed to render them less discrete and more difficult to conceal.

Optimism regarding the significant declines in e-cigarette use observed in the study by Gaiha et al^4^ may be tempered by the possibility that use will rebound to pre–COVID-19 levels as the COVID-19 pandemic eventually recedes. However, the findings from the study by Gaiha et al^4^ highlight several insights into how we might ensure that the declines remain durable. First, Gaiha et al^4^ suggest that youth were responsive to concerns regarding the health effects of vaping, especially in the context of risks for COVID-19 morbidity; thus, it is imperative that the evolving evidence on e-cigarette harms is widely disseminated through social media and other avenues. Second, as e-cigarette purchases shift to online sales, it is important to impose stronger safeguards, including more stringent age verification, while also taking measures to restrict access at conventional purchasing points, such as gas stations and vape shops. Third, to maintain the reductions that have occurred and to prevent relapse of youth who formerly vaped, it is essential that robust addiction services be put in place to provide the necessary services for youth struggling with nicotine addiction. Together, these measures may go a long way toward ensuring that the declines in e-cigarette use observed during the COVID-19 pandemic are maintained into the future.
